# Progress in Diabetes

**Published:** 1901-08-03

**Authors:** 


### Progress in Diabetes.

The morbid changes in the brains of diabetic patients, according to Howship Dickinson in his Baillie lectures,[1] are more in the nature of the transudation of blood round arteries than hæmorrhage due to ruptured vessels. He holds that this is due probably to an error in innervation of the blood-vessels rather than to the saccharine state of the blood, for it occurs in other diseases besides diabetes. This faulty innervation of the vessels may be the cause of the engorgement of the hepatic vessels. The capillary dilatation so-called is due, in his opinion, to post-mortem gaseous decomposition. He gives as the causes of the disease heredity and mental impressions, anxiety, fright, etc. This mental condition is most important. The loss of patellar reflex may be primary, and therefore nervous, though it may be due to the changes in the blood. Phosphaturia is most noticeable in diabetics, and this is also an accompaniment of perturbed state of the brain; while glycosuria has been noticed in cases of insanity. There is a larger percentage of loss of phosphate of lime than in health, and this probably has to do with mental and nervous waste.

With regard to the presence of sugar in the urine he offers [2] as a suggestion that normally the liver is restrained from passing glycogen into sugar, as in post-mortem changes, by the nerves: "this saccharine conversion needs to be bridled in two places, the liver holding one rein and the pancreas the other." With regard to treatment he does not put much faith in opium, but thinks that it may be harmful in producing what should be avoided as far as possible, viz., the obstinate constipation, and the control exerted upon the glycosuria is often counterbalanced by the invitation given to coma. He recommends strychnia in association with tartrate of potash (no precipitation) and phosphate of potash; but he lays stress on the large use of soft pure water.

The onset of coma in diabetes is to be feared according to Herter,[3] whenever the nitrogen of ammonia in the urine reaches 20 per cent. He attributes coma to the want of neutralisation of the acids in the blood by the ammonia, which would normally go to form urea. He holds that this is a preservative action upon the part of the body to keep down the "high acidity" of the blood.

Herter[4] maintains that the diabetic puncture acts by dilating the vessels of the liver to such an extent that the normal storing of carbohydrates cannot take place. He has seen the pancreas diseased in every post-mortem on diabetics he has attended; the chief affection being the compression of the cells in the Island of Langerhans by inflammatory connective tissue. Normal blood has the power of decomposing sugar, diabetic blood has lost much of that power. The appearance then of sugar in the urine is due to want of its combustion, and is closely related to the disease of the Island of Langerhans in the pancreas. He asserts that as far as prognosis is concerned the estimation of the amount of acid is more important than the amount of sugar excreted. The amount of acid passed is an indication that the amount of free alkali in the body has been used up. This alkali is mostly the ammonia obtained from food (meat), but when insufficient the cells themselves are depleted of their sodium salts, with the result that not sufficient carbonates remain to affect the interchange of gases, on which the tissues depend for their gain of oxygen and loss of carbonic-acid gas. Hence the cells are in a state of asphyxia. The kind of acid is immaterial for this to occur—it can be produced by hydrochloric or other mineral acids. This should prove an indication for the therapeutic use of sodium carbonate.

Coma has been produced again by Grube[5] by the injection into cats of $\beta$-amido-butyric acid. He holds that this body is the agent of coma in diabetes. The expressed juices of the liver, spleen and pancreas yield no reducing reaction upon grape-sugar solutions; but Umber[6] finds a distinct though small, reaction with blood. He finds no difference, however, in the specimens of blood taken from the different vessels of the body from that in the pancreatic vein.

The action of sodium salicylate has been studied by Williamson[7] in twenty cases of persistent glycosuria, and in most of them he records a marked diminution of the sugar excreted and an amelioration of other symptoms. In some the sugar disappeared while the drug was taken, but as soon as it was discontinued the sugar reappeared. He concludes that sod. salicylate is not a specific for diabetes; it does not usually produce any marked diminution of the sugar excretion in the severe forms of the disease, also it has little influence on some of the mild cases. It is not suitable to all cases, and its action must be carefully watched for toxic results. But in certain mild cases of diabetes or persistent glycosuria it has a decided action in very markedly diminishing the sugar excretion. Though the sugar in some cases is not reduced, yet the general condition and weight are improved.

Serum from the pancreatic vein of a dog was administered by the rectum in two cases of diabetes by Chatin and Guinard.[8] The authors conclude that the serum had no influence upon the disease. They conclude also that the internal secretion should be looked for in the lymphatics and not in the veins.

That sugar in the diet of diabetic patients may not be absolutely harmful is shown by Professor Lépine,[9] who cites a case of a diabetic who found that he gained strength by the addition to his diet of small quantities of sugar. Lépine suggests that small quantities of honey, consisting almost entirely of lævulose, the least harmful of the sugars, should be tried in different cases and the limit of tolerance found, which limit differs so much in different cases.

Glycosuria is met with in several pathological conditions—one case apparently produced by morphia taking is recorded by Keyser[10] from Marmaduke Shield's wards.

Heinrich Stern[11] writes of glycosuria being caused by excessive smoking of cigars. He states that people with a tendency to alimentary glycosuria will show it more readily after a saccharine meal followed by hard smoking than without the use of the tobacco, and the affection lasts longer. The lighter forms of this complaint may be changed to the more severe by the excessive use of tobacco; and there is evidence that this habit has a more baneful influence over the diabetic than on healthy people. The withdrawal of tobacco should be the treatment for those diabetics who suffer from certain disorders, as affections of the heart, eyes, and respiratory organs.

Out of 1,600 dyspeptic patients Robin found 5·2 per cent. glycosuric. In one set it was of the alimentary type. In a second set it was amenable to treatment, though convertible into true diabetes. Milk diet was sufficient treatment for some, others required "hepatic stimulation," with such drugs as sod. bicarb., sod. and pot. arsenites, codeine, belladonna, and valerian.[1] It was easily produced by Nobécourt[12] in rickety children by a quantity of glucose, which had no effect upon the healthy. In health the total daily amount of carbohydrates in urine should not exceed 5 grammes (average 1·6 gr.), but Rosin and V. Alfthan[13] have found that in diabetes mellitus the excretion is 9-20 grammes. Thus the disease shows a disturbance in all carbohydrate metabolism, not only glucose.

According to Edsall[13] the carbohydrates in diabetes insipidus are partly dependent upon the amount of fluid passed. A new reaction for sugar is described by Riegal.[14] He takes 0·1 grain of pure white hydrochloride of phenylhydrazin, 0·5 grain sod. acetate, and 2 c.c. of water; places these in a white porcelain crucible and boils with 20 drops of the suspected solution. If sugar be present even as small an amount as 0·005 per cent., it can be detected by a red violet colour being produced, which is rendered more apparent by the slow and cautious addition of 20–30m. of 10 per cent. soda solution.

In the nitro-propiol test for sugar it is recommended[15] to add the tablet of the reagent to the 10 c.c. of urine with 10 c.c. of water last, to prevent the formation of indigo white which may otherwise be formed. The indigo-blue colour is more easily recognised by shaking with a few drops of chloroform. It is said not to be given with the morbid urinary constituents and drugs such as excess of uric acid, iodides, etc. Large quantities of albumen, however, should be removed.

Eastes[16] urges that the phenylhydrazine test for sugar is especially valuable when the amount of sugar is so small as not completely to reduce Fehling's solution. If, after a minute's boiling, a greenish-yellow colour with some flocculence supervenes, then this test will be found to aid the diagnosis. Albumens should be eliminated first by boiling with a drop of acetic acid. If the partial reaction is again noticed then the 60 c.c. of the urine are evaporated on a water bath, with 1 gram sodic acetate and rather less phenyl hydrazine hydrochlorate, until 10 c.c. are left. With a glass rod the solid deposit must be scraped off as it occurs, so as to leave none of the sugar. With a microscope the crystals may be recognised in the cool fluid, and differentiation made between glucose and lactose by shape.

Walker Hall[16] gives as his experience that the best results are produced with 0·5 gram phenylhydrazine, 1·5 gram sod. acetate, and 5 c.c. of urine for glucose, but 10 c.c. should be used for maltose and lactose. This answers with 0·1 to 5 per cent. solutions.

For treatment of diabetes mellitus, Van Noorden[17] recommends Jambul, Aspirin, Carlsbad water and ordinary warm water, while the salts of ac. salicyl are specially recommended, and have also a beneficial action upon the pruritus. Bromides he gives for the insomnia and palpitation, etc., while opium he reserves for the grave neurotic disorders. Bismuth preparations are useful in diarrhœa and flatulence.

[1] Lancet, Feb. 2. [2] Lancet, Feb. 9. [3] Med. Rec., Jan. 19. [4] Med. Rec., Feb. 9. [5] Brit. Med. Jour., Dec. 8. [6] Brit. Med. Jour., Dec. 8. [7] Brit. Med. Jour., Mar. 30. [8] Brit. Med. Jour., Jan 12. [9] Brit. Med Jour., Jan. 5. [10] Lancet, Mar. 9. [11] Med. Rec., April 27. [12] Birm. Med. Rev., Nov. [13] Amer. Jour. Med. Sci., May. [14] Lancet, Feb. 2. [15] Lancet, April 6; Med. Notes, March 1900. [16] Brit. Med. Jour., Feb. 23. [17] Amer. Jour. Med. Sci., May.

